# Predicting Aggressive Tendencies by Visual Attention Bias Associated with Hostile Emotions

**DOI:** 10.1371/journal.pone.0149487

**Published:** 2016-02-22

**Authors:** Ping-I Lin, Cheng-Da Hsieh, Chi-Hung Juan, Md Monir Hossain, Craig A. Erickson, Yang-Han Lee, Mu-Chun Su

**Affiliations:** 1 Division of Biostatistics and Epidemiology, Cincinnati Children’s Hospital Medical Center, Cincinnati, Ohio, United States of America; 2 Department of Psychiatry and Behavioral Neuroscience, University of Cincinnati College of Medicine, Cincinnati, Ohio, United States of America; 3 Department of Computer Science and Information Engineering, National Central University, Taoyuan, Taiwan; 4 Institute of Cognitive Neuroscience, National Central University, Taoyuan, Taiwan; 5 Division of Child and Adolescent Psychiatry, Cincinnati Children’s Hospital Medical Center, Cincinnati, Ohio, United States of America; 6 Department of Electrical Engineering, Tamkang University, New Taipei, Taiwan; University of Tuebingen Medical School, GERMANY

## Abstract

The goal of the current study is to clarify the relationship between social information processing (e.g., visual attention to cues of hostility, hostility attribution bias, and facial expression emotion labeling) and aggressive tendencies. Thirty adults were recruited in the eye-tracking study that measured various components in social information processing. Baseline aggressive tendencies were measured using the Buss-Perry Aggression Questionnaire (AQ). Visual attention towards hostile objects was measured as the proportion of eye gaze fixation duration on cues of hostility. Hostility attribution bias was measured with the rating results for emotions of characters in the images. The results show that the eye gaze duration on hostile characters was significantly inversely correlated with the AQ score and less eye contact with an angry face. The eye gaze duration on hostile object was not significantly associated with hostility attribution bias, although hostility attribution bias was significantly positively associated with the AQ score. Our findings suggest that eye gaze fixation time towards non-hostile cues may predict aggressive tendencies.

## Introduction

Aggressive tendencies not only predict violent behaviors, but also self-injurious or suicidal behaviors [1,2]. Unraveling the mechanisms underlying aggressive tendencies may provide an initial step towards the development of innovative intervention strategies. Hostility attribution bias (i.e., over-interpretation of others’ behaviors as hostile), a type of biased social information processing, has been found to be associated with the risk of aggressive behaviors [3]. Social information processing includes several steps, of which the reception and coding of social stimuli are largely based on viewing facial expressions of other individuals. Aggressive tendencies have been shown to positively correlate with the likelihood of labeling neutral or ambiguous facial expressions as angry ones, regardless of the intellectual capacity [4–6]. Individuals with anti-social behaviors have been shown to have the tendency to misinterpret nonverbal cues in social interactions [7]. A better understanding of the mechanisms underlying hostility attribution bias may hold the key to informing intervention strategies for aggressive behaviors.

Cognitive distortions have been thought to play a role in hostility attribution bias [8]. Mood dysregulation has been found to contribute to self-directed hostility attribution [9,10]. Specifically, cognitive distortions associated with hostility attribution bias may include paranoid thinking and suspicions [11]. Additionally, cognitive distortions, such as memory bias towards angry faces, have been found to be more prominent in impulsive aggressive adolescents [12]. On the other hand, perceptive social information processing bias, such as selective visual attention towards hostile objects, may play a role in hostility attribution bias [8]. One study reports that attention bias towards overtly hostile objects was associated with neither aggressive tendencies nor hostility attribution bias in children without mental disorders [13]. On the contrary, another study reported that children with oppositional defiant disorder, which is characterized by persistent pattern of tantrums, arguing, and angry or disruptive behavior toward, had less eye contact with their mothers, compared to healthy controls [14]. However, children with bipolar disorder and co-morbid anxiety disorders are found to exhibit attention bias towards threat faces [15]. It remains arguable regarding whether visual attention bias towards hostile objects may contribute to hostility attribution bias. In the current study, we examined if aggressive tendencies could be associated with two cognitive correlates associated with hostility attribution bias: labeling facial expression emotions and selective visual attentions.

## Methods

### 1. Subject recruitment

A total of 36 young adults (age range: 18–30 years) were recruited for this study. The exclusion criteria include (1) a history of psychotic disorders, (2) a history of depressive or bipolar disorder, and (3) a history of substance use disorders. We used the self-reported questionnaire to exclude the individuals with these four types of mental disturbances.

### Statement of ethics

Twenty-five of the 36 participants were recruited through on-line advertisement, and provided their consents by signing in the on-line spreadsheet. Another 11 participants were enrolled through the biorepository at Cincinnati Children’s Hospital Medical Center (CCHMC). This biorepository project has been conducted based on the protocol approved by Institutional Review Board (IRB) of CCHMC. The individual in this manuscript has given written informed consent (as outlined in PLOS consent form) to publish these case details.

### 2. Data acquisition

#### 2.1. Aggressive tendencies

We used Buss-Perry Aggression Questionnaire (AQ) to assess the baseline aggressive tendencies [16]. The questionnaire is composed of 29 items. Each item is a 7-point continuum scale (1 = totally disagree, 5 = totally agree). AQ allows participants to rank their own characteristics for each statement related to four categories: physical aggression, verbal aggression, hostility, and anger. The scales show internal consistency and stability over time. The various scales correlated differently with various personality traits. The test-retest reliability of this questionnaire was 0.78 [17]. In order to adjust for the confounding effect of stress, we also asked each participant to fill out the 10-item Perceived Stress Scale (PSS) [18], so that the perceived stress level could be adjusted.

#### 2.2. Eye tracking tests

*Stimuli*: We have designed a series of pictures to present embedded ambiguous signs of hostility in the context of social interactions to each participant. These social interactions were classified into two categories: “confrontational” and “non-confrontational.” During the confrontational session, a series of 10 pictures of facial expressions retrieved from MMI Facial Expression Database [19] were presented to each participant. The viewing time was fixed at 20 seconds for each image. The stimuli were used to mimic the situation, in which the participant would be engaged in direct eye contacts with another person staring at him or her for 20 seconds. During the non-confrontational session, each participant was instructed to view 7 pictures of interactions between two or three individuals. The viewing time for each picture was also 20 seconds. The participant was also required to answer questions to interpret the intentions of characters in the picture. These stimuli were used to mimic the situation that each participant acted as a bystander at events that involved ambiguous inter-personal conflicts.

*Eye tracking experiment*: Eye gaze patterns were recorded using the Eyelink II and analyzed using Eye Link^®^ Data Viewer (SR Research Ltd., Mississauga, Ontario, Canada) according to the user’s manual. The area of interest (AOI) was determined by the research team to concentrate on the regions that were hypothesized to represent the target of social information processing. For images related to social interactions, the AOIs were defined as regions including the face of each character and other body parts that intuitively referred to the body language (e.g., waving hands). For images of individual facial expressions, the AOIs included eyes, noses, and mouths, which were found to more frequently viewed than the other parts of the face [20].

### 3. Statistical methods

Under the assumption of effect size f^2^ = 0.2, and alpha-value = 0.05, we estimated that we would achieve the power of 0.71 with the sample size equal to 36. Therefore, our sample size might be adequate given that we aimed to obtain a moderate statistical power. The primary predictor variable was the eye gaze fixation time at each AOI. All key continuous variables (i.e., AQ scores, eye gaze fixation times, and PSS scores) were normalized with inverse rank-based transformation before the further analysis. We used the step-wise regression model to select significant independent variables to partially remedy under-estimated coefficients due to multicollinearity. The relationship between aggressive tendencies (AQ scores) and eye gaze fixation times was examined using the general linear model with PSS scores, gender, and age being adjusted as covariates. The area of AOI was adjusted in the regression model. Two-sided alpha value = 0.05 was used as the cut-off to screen for significant association findings. We later performed single-value permutation tests (N = 1,000) to generate empirical p-values in order to alleviate inflated type-I errors due to multiple tests. We also test spatial homogeneity for eye gaze fixation points in the AOI with Ripley’s K functions. The measure of the degree of spatial homogeneity was used to examine whether the degree of “attention dispersion” correlated with the AQ score. Attention dispersion might be regarded as another aspect of visual attention.

## Results

**Fig 1:**
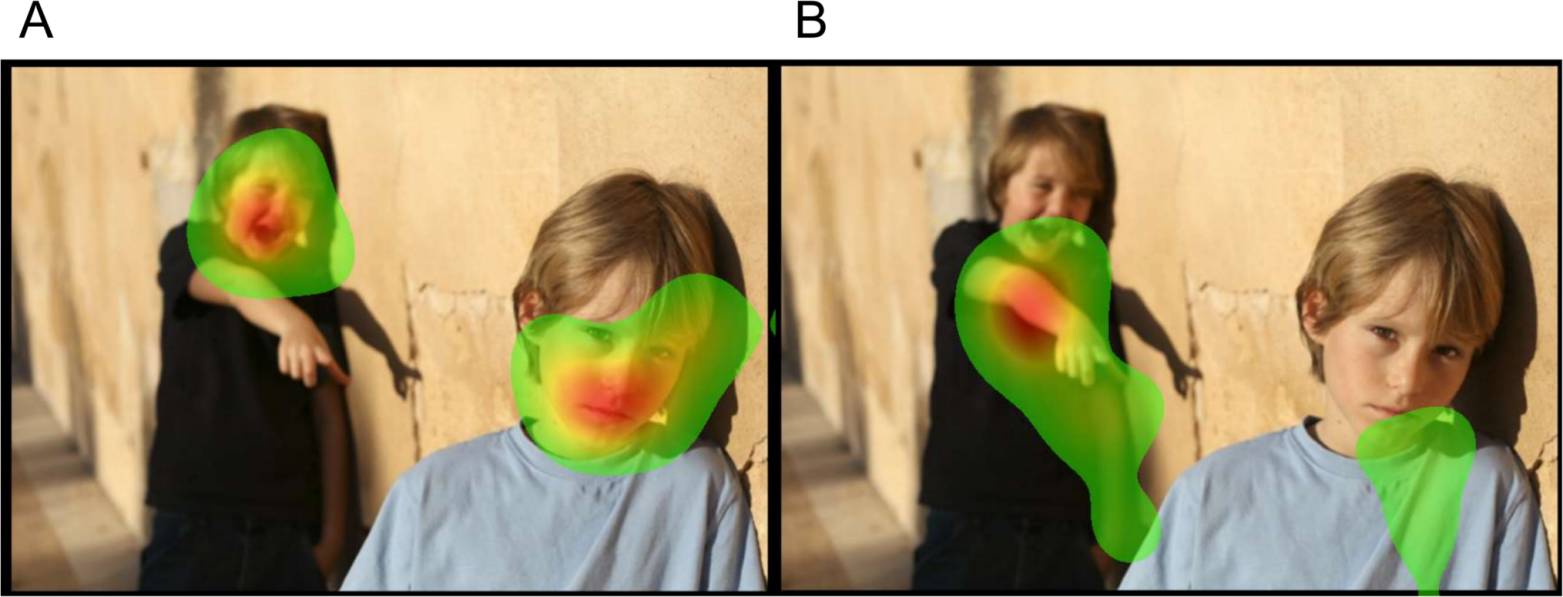
Heatmap of visual attentions towards images of “non-confrontational” social interactions is shown. A is the individual with AQ score at the 25^th^ percentile. B is the individual with AQ score at the 75^th^ percentile. The eye gaze fixation time is indicated by color, of which the order of duration is red > yellow > green.

**Fig 2:**
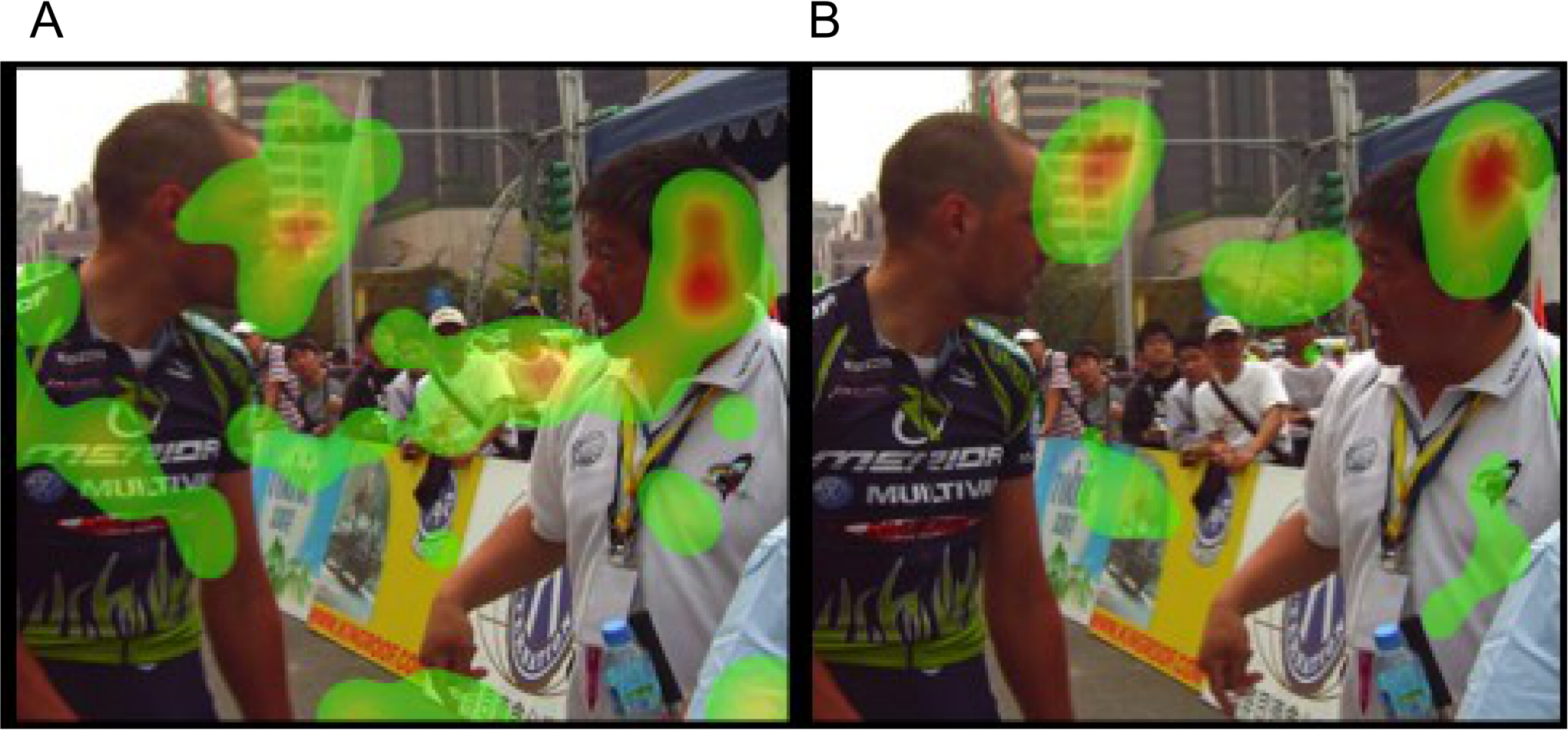
Heatmap of visual attentions towards images of “confrontational” social interactions is shown. A is the individual with AQ score at the 25^th^ percentile. B is the individual with AQ score at the 75^th^ percentile. The eye gaze fixation time is indicated by color, of which the order of duration is red > yellow > green.

**Fig 3:**
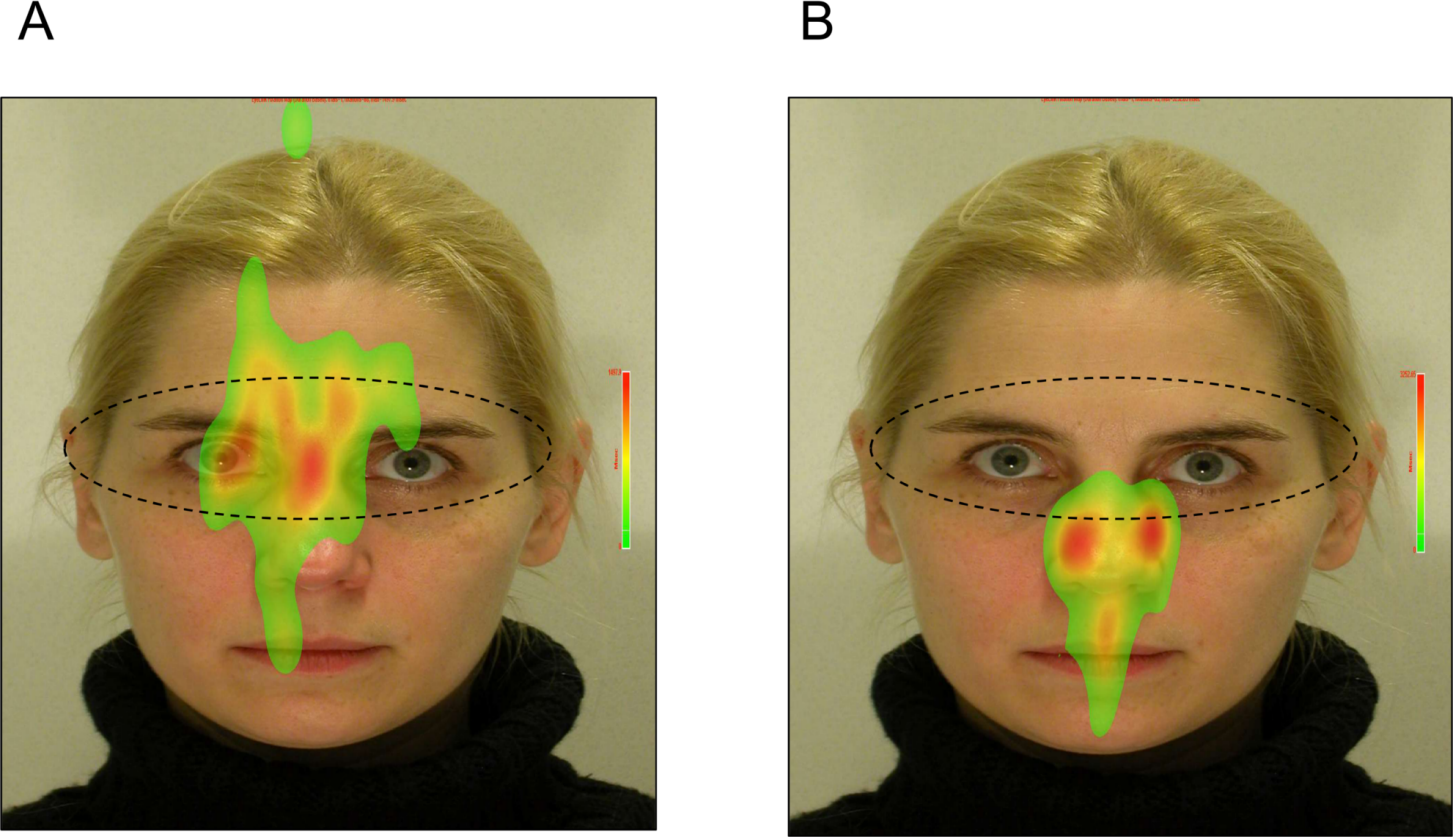
Heatmap of visual attentions towards images of angry facial expressions is shown. A is the individual with AQ score at the 25^th^ percentile. B is the individual with AQ score at the 75^th^ percentile. The eye gaze fixation time is indicated by color, of which the order of duration is red > yellow > green.

Table 1 summarizes the distributions of each variable of interest. AQ scores were significantly correlated with all 4 categories (i.e., physical aggression, verbal aggression, anger, and hostility) (Pearson correlation test, p < 0.0001), and hence we only examined the relationship between the summary AQ score and key predictors. Table 2 summarizes the relationship between selective visual attentions and aggressive tendencies. The analyses with the general linear model show that AQ scores were significantly associated with eye gaze fixation time on non-facial body parts (e.g., pointing fingers) of the character who showed more hostile emotions in the context of non-confrontational social interactions (coefficient = 0.02, F = 5.24, permuted p-value = 0.031). However, the similar relation was not significant in the context of confrontational social interactions. AQ scores were also significantly associated with eye gaze fixation time on the mouth areas in the facial expression images (coefficient = 0.01, F = 10.69, permuted p-value = 0.005). Fig 1 shows the heatmap for eye fixation patterns of the two participants viewing the images of “non-confrontational” social interactions. AQ scores, and Fig 2 shows the heatmap for eye fixation patterns of the two participants viewing the images of “confrontational” social interactions. Fig 3 shows the heatmap for eye fixation patterns of the two participants viewing the image of the facial expression. Furthermore, we found that “attention dispersion” was also nominally significantly associated with AQ scores (permuted p-value = 0.01) at the same AOIs as those shown in the fixation time analysis.

**Table 1:** Demographic features of study participants.

Variable	Mean	SD	Min	Max
AQ	94.76	21.84	57	140
Physical aggression	27.24	8.35	14	48
Verbal aggression	17.60	5.82	8	31
Anger	21.52	5.67	13	34
Hostility	28.4	9.19	11	45
Age*	22.16	2.66	19	32
	Male	Female		
Sex*	72%	28%		

* Not significantly associated with any factor of aggressive tendencies.

**Table 2:** The relationship between selective visual attentions and aggressive tendencies.

Selective visual attention to images of social interactions			
	Coefficient	F statistics	Permuted p-value
Confrontational	-0.002	0.107	>0.05
Non-confrontational	0.02	5.24	0.031
Selective visual attention to images of facial expressions			
	Coefficient	F statistics	Permuted p-value
Neutral/Ambiguous	0.0002	0.03	>0.05
Angry	0.01	10.69	0.005

## Discussion

Our findings suggest that selective visual attention towards objects with hostility may be associated with aggressive tendencies in young adults. Specifically, we found that individuals with greater aggressive tendencies seemed to pay more visual attention to the non-facial parts of the hostile character in the context of social interactions, compared to individuals with lower aggressive tendencies. This may lend little support to the hypothesis that selective visual attention to hostile objects can contribute to aggressive tendencies. Similarly, we found that individuals with greater aggressive tendencies seemed to pay more visual attention to non-eye regions of the angry face, compared to their peers with lower aggressive tendencies. This may imply that individuals with greater aggressive tendencies may show less eye contact when being faced with possible hostility, compared to their peers with lower aggressive tendencies. Taken together, aggressive tendencies appear to be inversely correlated with eye contact in response to the object of hostility. The association appears to be more remarkable when the individual interacts with another hostile individual than serving as a by-stander in the scene of social conflict.

Eye contact indicates several different kinds of social communications. Previous studies have suggested that eye contact aversion often occurs in an aggressive encounter [21]. In primates, individuals usually show their aggressiveness by staring, while attempt to reduce conflicts by averting the gaze [22]. Therefore, our findings suggest that aggressive individuals may be more likely to reduce such conflicts than non-aggressive individuals. Nevertheless, eye gaze aversion in an aggressive individual may also indicate a defense mechanism, in which anxiety-producing emotions and impulses are masked by exaggeration of the directly opposing tendency. Additionally, our findings did not support the hypothesis that aggressive individuals pay more direct attention to hostile objects, but pay more attention to “cues” of hostility (e.g., fingers pointing to the child being verbally bullied in Fig 1). Therefore, our findings may not rule out that aggressive tendencies are associated with selective visual attention towards hostility, because the attention bias may only be observed at specific symbols of aggression/hostility.

To evaluate if the data based on early short time-windows were different from the current results, we re-examined the eye gaze fixation points during the first 5 seconds. The results show that there was no statistically significant correlation between aggressive tendencies and region-specific fixation time. Our explanation is that the visual stimuli in the first 5 seconds might be primarily driven by immediate emotional response, which might not be able to sufficiently distinguish individuals with higher aggressive tendencies from individuals with lower aggressive tendencies. When the viewing time is extended, how visual attention is sustained towards certain regions might be able to distinguish individuals with higher aggressive tendencies from individuals with lower aggressive tendencies.

The primary limitation of the current study is the sample size. The age range of our sample (>80% of individuals aged between 20 and 25 years) and characteristics of participants (volunteer college students) may also discourage the generalizability of our findings. In addition, we did not perform stringent multi-testing corrections, such as Bonferroni method, and hence inflated type-I errors may remain to be a problem. However, we did perform single-value permutations to generate empirical p-values to identify significant associations, so inflated type-I errors may be partially alleviated. Finally, we relied on self-reported information to exclude subjects with mental disorders. The impact of under-reported cases of mental disorders may lead to uncertain directions of bias on our findings.

Several recent studies also report neural correlates or eye gaze patterns in individuals viewing violent conflicts. Briefly, how an individual reacts to stressful stimuli (e.g., fearful or threatening faces) may be affected by several different factors, such as affective information in the background [23,24]. Furthermore, a previous study suggests that personality traits, such as dominance, may be associated with a slower “gaze aversion” when focusing on aggressors in a violent conflict [25]. These recent findings indicate that the differences in personality traits and emotional responses to violent conflicts are modulated by activations in different brain regions. In the current study, we found that individuals with greater aggressive tendencies might exhibit less gaze fixation time towards eye regions of both aggressors and victims, but there was a trend (which was not statistically significant) that more aggressive individuals might also sustain longer visual attention to angry body postures when viewing a violent conflict. We believe that the differences between our findings and these recent reports are due to the difference in study designs, such as measurements of aggressive tendencies and visual stimuli. In our study, we focused on relatively more ambiguous violent contexts. Additionally, we used Buss-Perry Aggression Questionnaire to measure aggressive tendencies–of which the scores have not been shown to correlate with levels of dominance.

In conclusion, we have found that selective visual attention associated with hostile emotions might serve as a physiological/behavioral marker for aggressive tendencies in non-mentally ill individuals. Since aggressive tendencies may predict aggressive and violent behaviors, selective visual attention towards objects of hostility may also predict aggressive or violent behaviors. Replication of these findings in a larger population is needed to evaluate the clinical utility of eye gaze fixation time (a proxy of visual attention bias) in at-risk individuals. The findings may inform early detection and prevention modalities of aggressive/violent behaviors in the future. Previous evidence suggests that the patterns of visual attention bias towards hostility in healthy individuals seem to depend on the diagnosis of mental disorders. Further research is warranted to compare the cognitive and behavioral mechanisms underlying aggressive tendencies across different diagnoses.

## Supporting Information

S1 Dataset Minimal dataset of eye gaze fixation time (nine areas of interest in three subjects). (DOCX)
