# Supplementary material for: Predicting Aggressive Tendencies by Visual Attention Bias Associated with Hostile Emotions
Source: PLoS One. 2016 Feb 22;11(2):e0149487. doi: 10.1371/journal.pone.0149487 (PMC4763877; doi:10.1371/journal.pone.0149487)
Supplement: S1 Dataset — (DOCX) [file pone.0149487.s001.docx]

Dataset S1. Minimal dataset of eye gaze fixation time (nine areas of interest in three subjects)

Areas of interest

| ID | ar1 | ar2 | ar3 | ar4 | ar5 | ar6 | ar7 | ar8 | ar9 |
| --- | --- | --- | --- | --- | --- | --- | --- | --- | --- |
| a1 | 180 | 120 | 416 | 660 | 940 | 852 | 1464 | 688 | 1252 |
| a2 | 0 | 0 | 0 | 504 | 0 | 524 | 1636 | 956 | 240 |
| a3 | 320 | 1088 | 120 | 1832 | 2824 | 1420 | 796 | 748 | 2736 |
